# Celebrating Professor Rajeev K. Varshney's transformative research odyssey from genomics to the field on his induction as Fellow of the Royal Society

**DOI:** 10.1111/pbi.14282

**Published:** 2024-01-11

**Authors:** Vanika Garg, Rutwik Barmukh, Annapurna Chitikineni, Manish Roorkiwal, Chris Ojiewo, Abhishek Bohra, Mahendar Thudi, Vikas K. Singh, Himabindu Kudapa, Rachit K. Saxena, Jake Fountain, Reyazul Rouf Mir, Chellapilla Bharadwaj, Xiaoping Chen, Liu Xin, Manish K. Pandey

**Affiliations:** ^1^ Centre for Crop & Food Innovation, WA State Agricultural Biotechnology Centre, Food Futures Institute Murdoch University Murdoch Western Australia Australia; ^2^ Khalifa Center for Genetic Engineering and Biotechnology United Arab Emirates University Al Ain UAE; ^3^ International Maize and Wheat Improvement Center (CIMMYT) Nairobi Kenya; ^4^ Fort Valley State University Fort Valley Georgia USA; ^5^ International Rice Research Institute (IRRI)‐South‐Asia Hub International Crops Research Institute for the Semi‐Arid Tropics Hyderabad India; ^6^ Center of Excellence in Genomics & Systems Biology International Crops Research Institute for the Semi‐Arid Tropics (ICRISAT) Hyderabad India; ^7^ Gujarat Biotechnology University (GBU) Gandhinagar India; ^8^ Department of Plant Pathology University of Georgia Griffin Georgia USA; ^9^ Division of Genetics and Plant Breeding, Faculty of Agriculture SKUAST‐Kashmir Wadura India; ^10^ Division of Genetics ICAR‐Indian Agricultural Research Institute New Delhi India; ^11^ Crops Research Institute Guangdong Academy of Agricultural Sciences (GDAAS) Guangzhou China; ^12^ BGI Research Beijing China

**Keywords:** agricultural biotechnology, crop genomics, plant breeding, agriculture, genome sequencing

## Abstract

Professor Rajeev K. Varshney's transformative impact on crop genomics, genetics, and agriculture is the result of his passion, dedication, and unyielding commitment to harnessing the potential of genomics to address the most pressing challenges faced by the global agricultural community. Starting from a small town in India and reaching the global stage, Professor Varshney's academic and professional trajectory has inspired many scientists active in research today. His ground‐breaking work, especially his effort to list orphan tropical crops to genomic resource‐rich entities, has been transformative. Beyond his scientific achievements, Professor Varshney is recognized by his colleagues as an exemplary mentor, fostering the growth of future researchers, building institutional capacity, and strengthening scientific capability. His focus on translational genomics and strengthening seed system in developing countries for the improvement of agriculture has made a tangible impact on farmers' lives. His skills have been best utilized in roles at leading research centres where he has applied his expertise to deliver a new vision for crop improvement. These efforts have now been recognized by the Royal Society with the award of the Fellowship (FRS). As we mark this significant milestone in his career, we not only celebrate Professor Varshney's accomplishments but also his wider contributions that continue to transform the agricultural landscape.

## Introduction

**Figure 1:**
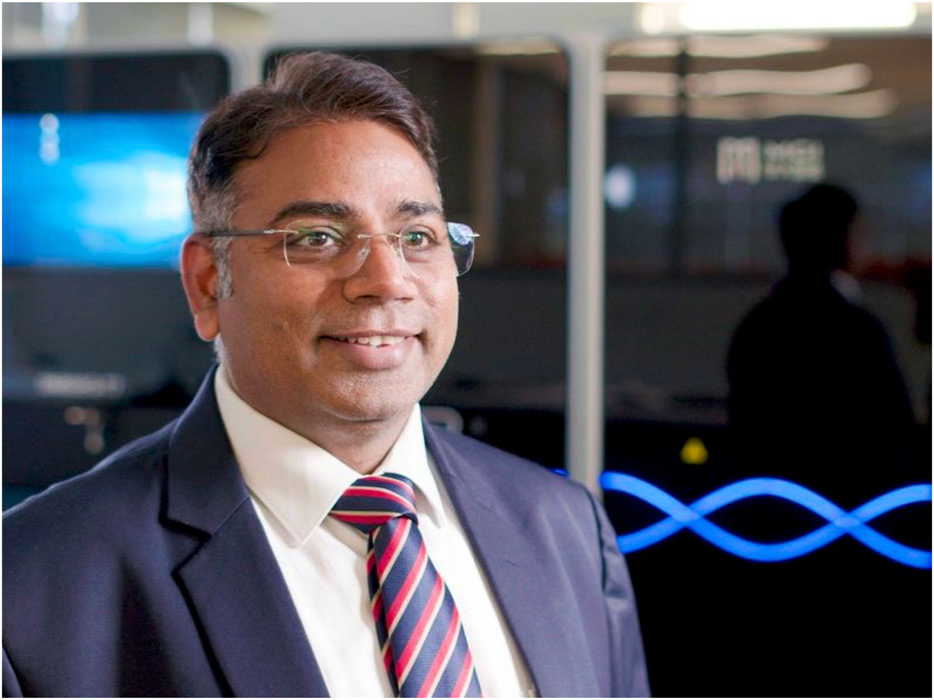
Professor Rajeev K Varshney in his element at WA State Agricultural Biotechnology Centre/Centre for Crop & Food Innovation, Murdoch University in 2023.

**Figure 2:**
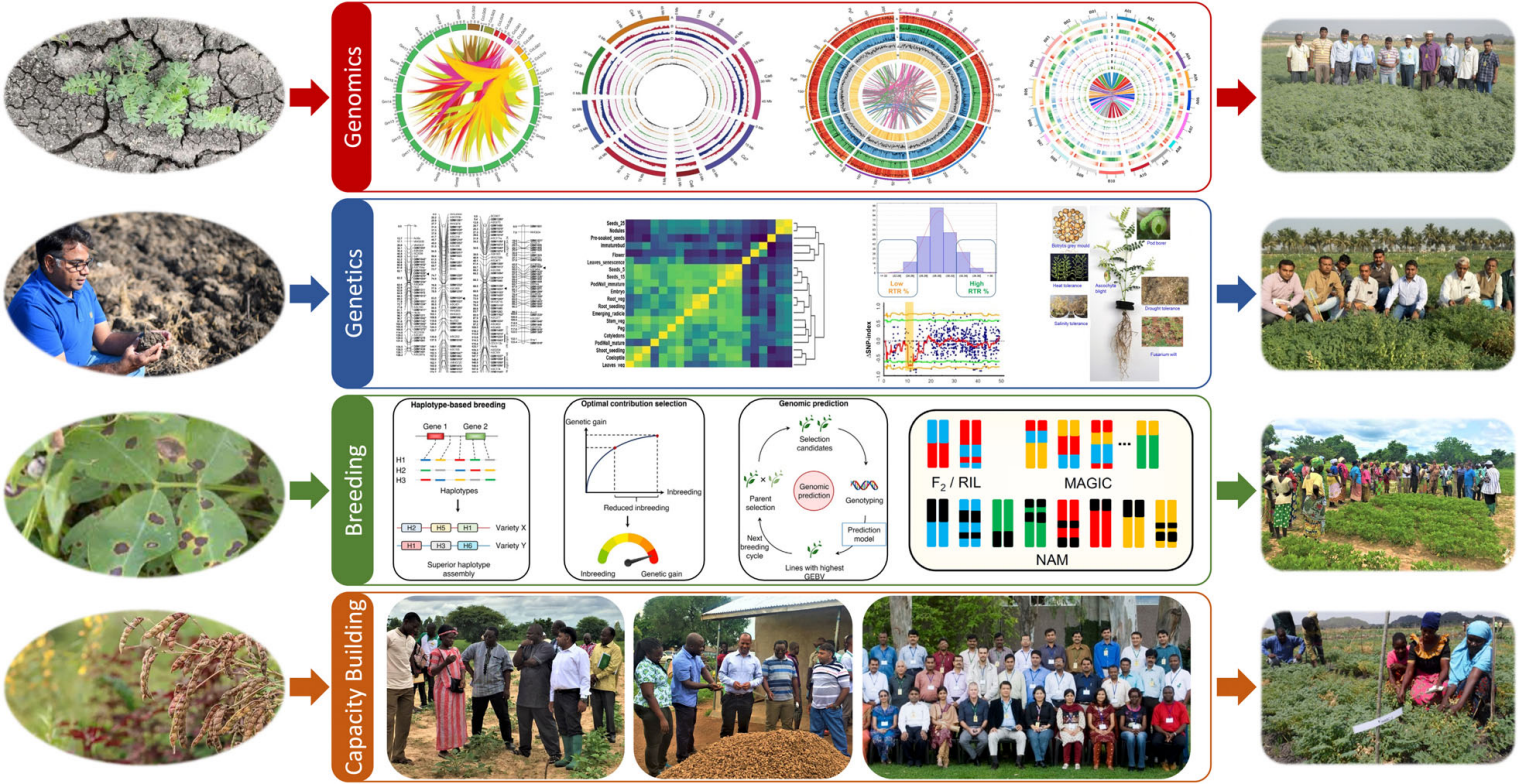
Professor Varshney's pioneering contributions in agricultural genomics applied to crop improvement. Professor Varshney's global leadership is underscored by his extensive work on genome sequencing, genetic diversity cataloguing, genomics‐assisted breeding, seed systems enhancement, capacity‐building, and empowering national programmes in developing countries. His research journey embodies collaborative excellence, rigorous genetic evaluations, and innovative breeding strategies, all aimed at transforming scientific breakthroughs into tangible advancements by delivering superior crop varieties to farmers worldwide.

Professor Rajeev Kumar Varshney (Box 1, Figure 1), popularly known by his colleagues as the “Genomics Guru”, is globally recognized leader for his work on genome sequencing, harnessing genetic diversity, genomics‐assisted breeding, seed systems, and capacity building in developing countries (Figure 2). Over his extensive research career spanning more than two decades, he has made significant contributions to improving food security in Asia and Africa by creating genomic resources and utilizing them for crop improvement in some major “orphan” tropical crops. Professor Varshney is deeply committed to equipping breeders with the tools and resources for genomics‐assisted breeding, including the provision of training in cutting‐edge techniques and enabling access to affordable genotyping and sequencing technologies. Furthermore, he has been the spearhead for major international initiatives that have delivered genomic resources and superior crop varieties for chickpea, pigeonpea, and groundnut to some of the world's poorest farmers.

Box 1 **Life sketch**

Born on 13 July 1973 in Bahjoi, Uttar Pradesh, India, Professor Varshney has established himself as a prominent figure in the realm of plant genomics, genetics, and transformative agriculture. He began his educational pursuit at Aligarh Muslim University, Aligarh, Uttar Pradesh, India, where he completed his B.Sc. Honours in Botany in 1993 and furthered his education with an M.Sc. in Botany, specializing in Genetics, Plant Breeding, and Molecular Biology in 1995. Professor Varshney pursued his doctoral studies under the aegis of Professor PK Gupta and Professor PC Sharma at Chaudhary Charan Singh University, Meerut, Uttar Pradesh, India. In 2001, he earned his PhD in Agriculture (Molecular Biology) for his notable work on a Wheat Biotechnology Project supported by the Department of Biotechnology, Government of India, with his thesis titled “A Study of Microsatellites in Hexaploid Wheats”. After completing his doctoral studies, he undertook the role of Wissenschaftlicher Mitarbeiter (Research Scientist) at the Leibniz Institute of Plant Genetics & Crop Plant Research (IPK), Gatersleben, Germany, under the mentorship of Professor Andreas Graner. During his stay at IPK for 5 years, he was deeply engaged in barley genomics research and comparative genomics across cereal crops. His exceptional skill set caught the attention of the International Crops Research Institute for the Semi‐Arid Tropics (ICRISAT), India, leading to his appointment as Senior Scientist for Applied Genomics in 2005. With a vision to catalyse genomics research in dryland crops, Professor Varshney, as Founding Director, spearheaded the Center of Excellence in Genomics in 2007 with the support of the Department of Biotechnology, Government of India. In 2017, this centre, under leadership of Professor Varshney, evolved into the Center of Excellence in Genomics & Systems Biology. During his tenure at ICRISAT, Professor Varshney occupied numerous esteemed roles, such as Principal Scientist ‐ Applied Genomics (2008–2013), SubProgramme Leader ‐ Consultative Group on International Agricultural Research (CGIAR)'s Generation Challenge Program, CIMMYT, Mexico (2007–2013), Global Research Program Director ‐ Grain Legumes (2013–2016), Global Research Program Director ‐ Genetic Gains (2016–2021), and Global Research Program Director Accelerated Crop Improvement (2021–2022). Professor Varshney's significant contributions reached a plateau when he developed invaluable genomic resources for tropical crops. His exceptional work made him a sought‐after scientist, and he was headhunted by several universities/institutes from the USA, the UK, Australia, Canada, and China. In 2022, Professor Varshney's illustrious career took him to Murdoch University, Australia, where he now holds multiple directorial positions (Director of the Western Australian State Agriculture Biotechnology Centre (SABC), Centre for Crop & Food Innovation (CCFI), and International Chair in Agriculture and Food Security with the Food Futures Institute) that emphasize advancements in agricultural biotechnology and fortify food security initiatives. Alongside his professional endeavours, Professor Varshney finds joy and support in his personal life with his wife, Monika Varshney, and their two children, Prakhar Varshney and Preksha Varshney.

Recognizing these efforts Professor Varshney was recently (May, 2023) elected as a Fellow of the Royal Society (UK). In the esteemed 366‐year history of the Royal Society, he is the fourth individual from India to be elected from the field of Agricultural Sciences and Forestry, and one of just 13 worldwide. Additionally, he is the fifth from Western Australia to be elected from any discipline. As a tribute to Professor Varshney's and his career, this article traces his remarkable journey from the early days of resolving genetic polymorphism on gels, to cataloguing genome diversity and advancing plant biology, and finally to his recent accolades, thereby celebrating a life committed to pushing the boundaries of scientific exploration. As we, some of his former PhD students, post‐docs, colleagues, and collaborators, look back over his career, we hope not only to appreciate the scientific accomplishments of Professor Varshney but also to draw inspiration from the spirit of perseverance and vision he embodies.

## Early influences and beginnings

**Figure 3:**
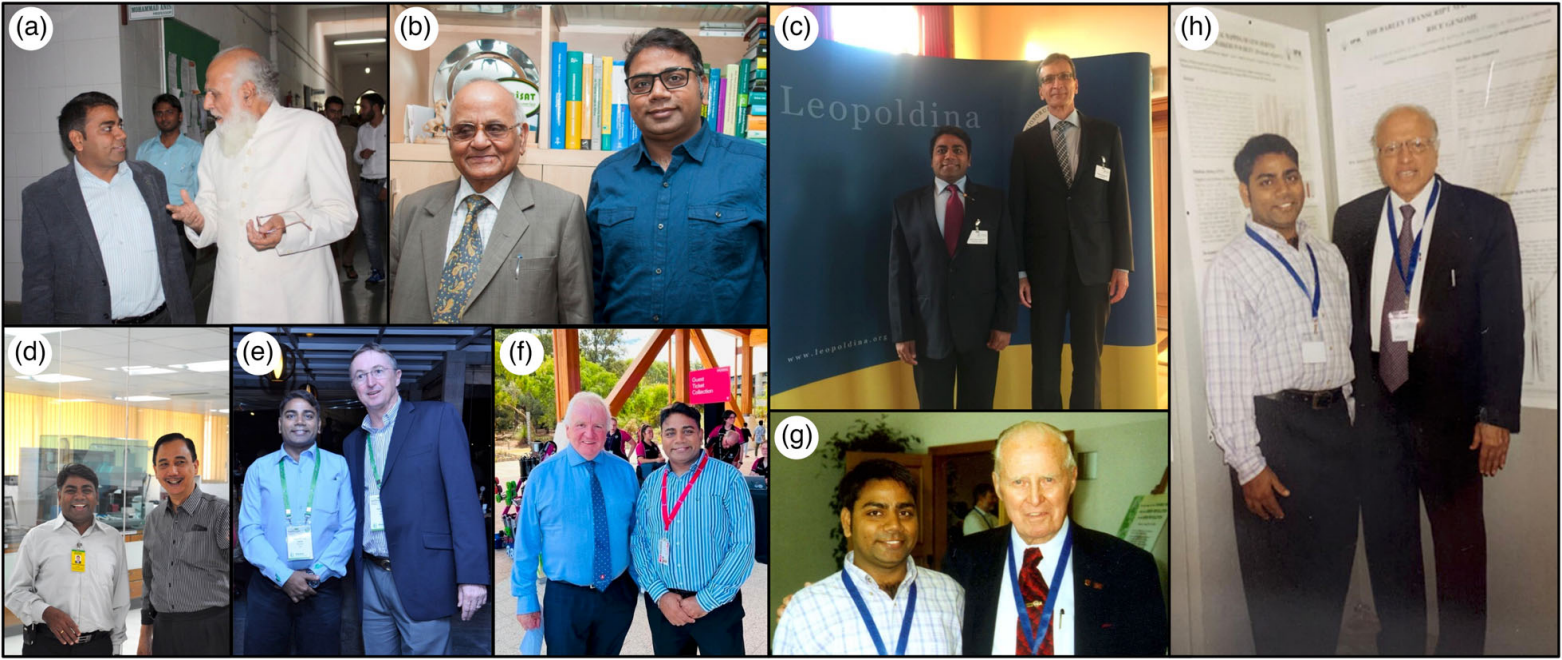
Key moments from Professor Varshney's esteemed career in the presence of distinguished individuals who have contributed to his professional journey. The snapshots showcase interactions with mentors and globally renowned scientists, reflecting the camaraderie and inspiration he has drawn throughout his distinguished research career. Professor Varshney with (a) Professor Wazahat Hussain (teacher during Bachelor's and Master's course); (b) Professor PK Gupta (PhD Supervisor); (c) Professor Andreas Graner (Post‐doc Supervisor); (d) Dr William Dar (former Director General, ICRISAT); (e) Dr Peter Carberry (former Director General, ICRISAT); (f) Professor Peter Davies (Pro Vice Chancellor & Director, Food Futures Institute, Murdoch University); (g) Professor Norman Borlaug, Nobel Laureate for Green Revolution; and (h) Professor MS Swaminathan, father of Indian Green Revolution.

Growing up in the agrarian landscapes of Bahjoi, Uttar Pradesh, India, Professor Varshney's education at Aligarh Muslim University in Uttar Pradesh, India, shaped his understanding of Botany, emphasizing genetics, plant breeding, and molecular biology. During his childhood and early student life, Professor Varshney was inspired by observations of smallholder farmers battling climatic and agronomic challenges, and generated an interest to do research for improving agriculture. Given his early interest in plant genetics, Professor Varshney pursued his PhD under the mentorship of distinguished geneticist Professor PK Gupta. Professor Gupta's example of hard work, commitment, and dedication established the foundations for Professor Varshney's future success (Figure 3).

Following the completion of his PhD, Professor Varshney started his post‐doctoral research at IPK, Gatersleben (Germany) under the mentorship of Professor Graner who instilled a passion for research, and the importance of creating a nurturing and supporting environment for all colleagues and collaborators, qualities that later became inherent to Professor Varshney's working style. It was during this time that Professor Varshney attended a scientific conference in Bologna (Italy) in 2003, where he heard a speech by Professor Norman Borlaug. Professor Borlaug's compelling message urging the next generation of scientists to create innovative and sustainable solutions to safeguard food security for the world's poor resonated deeply with Professor Varshney. Inspired by Professor Borlaug's vision, and motivated by his interactions with stalwarts of the Indian Green Revolution, including Professor MS Swaminathan, Professor Gurdev Khush, and other eminent scientists like Professor Mike Gale (UK) and Professor Ronald Philipps (USA), Professor Varshney made the decision to go back to India and join ICRISAT, a CGIAR global institute, with the intent of applying the knowledge and expertise he had gained in Europe to advance agriculture in the developing world. Professor Varshney acknowledges the invaluable mentorship and inspiration he received from both Professor Swaminathan and Professor Khush. Notably, Professor Swaminathan imparted critical insights on the importance of interdisciplinary research and collaboration in tackling the multifaceted challenges inherent in agriculture. These pivotal experiences and insights played a decisive role in shaping Professor Varshney's approach during his 17‐year tenure at ICRISAT and continue to inspire his work at Murdoch University, Australia.

## Research contributions

### Genomic resources in wheat and barley

Staple crops like wheat and barley, play a pivotal role in providing energy and protein for the rapidly growing global population. Professor Varshney's doctoral studies under the supervision of Professor PK Gupta primarily focused on enhancing the genetic and genomic resources i.e. development of microsatellte markers and their use for trait mapping in wheat to support marker‐assisted breeding programs (Gupta and Varshney, 2000; Kota *et al*., 2001; Varshney *et al*., 2000a,b, 2001). During his time at IPK with Professor Graner, he played a significant role in the development of various genomic resources for barley, including expressed sequence tags (ESTs), molecular markers such as EST‐SSRs, EST‐SNPs, high‐density genetic maps, and a transcript map (which involved integrating genes or transcripts with the genetic map) (Thiel *et al*., 2003; Varshney *et al*., 2004, 2005a,b, 2006a,b,c). Additionally, he contributed to the generation of a physical map of barley (Varshney *et al*., 2005a). These genomic resources were utilized for mapping Quantitative Trait Loci (QTLs) for agronomic traits through linkage mapping and association mapping approaches, conducting comparative studies in cereals and functional genomics studies for drought tolerance and malting quality in barley. These resources provided a springboard for sequencing the barley genome at IPK.

### Genome sequences of tropical crops

The pivotal role of genome sequencing in agriculture and crop improvement is now established. The genome sequence acts as the genetic roadmap for an organism, illuminating biological intricacies and evolutionary history, while also creating avenues for crop improvement. Recognizing the transformative power of genome sequencing, Professor Varshney began work to unlock the genetic secrets of crops crucial for food security and sustainable agriculture. In doing so, he led and/or contributed to the sequencing of more than 15 crop genomes, including chickpea (*Cicer arietinum* L.; Varshney *et al*., 2013), pigeonpea (*Cajanus cajan*; Varshney *et al*., 2012), diploid progenitor species (*Arachis duranensis*, Chen *et al*., 2016, and *A. ipaensis*, Bertioli *et al*., 2016), and cultivated species (*A. hypogaea*) of groundnut (Zhuang *et al*., 2019), pearl millet [*Cenchrus americanus* (L.) Morrone, Varshney *et al*., 2017a], sesame (*Sesamum indicum* L., Wang *et al*., 2014), mungbean (*Vigna radiata*, Kang *et al*., 2014), adzuki bean (*Vigna angularis*, Yang *et al*., 2015), longan (*Dimocarpus longan*, Lin *et al*., 2017), jatropha (*Jatropha curcas*, Ha *et al*., 2019), soybean (*Glycine max*; Garg *et al*., 2023a), celery (*Apium graveolens* L.; Song *et al*, 2021), and pea (*Pisum sativum*, Yang *et al*., 2022), with several others currently in progress.

In 2012, Professor Varshney decoded the genome sequence of pigeonpea (Asha genotype). This landmark effort marked pigeonpea as the first orphan legume crop genome to be sequenced, second only to soybean in food legumes. By integrating the next‐generation sequencing technologies with traditional bacterial artificial chromosome end sequences (BAC) based resources and a genetic map, his team mapped ~605.78 Mb of the 833.07 Mb genome and identified 48 680 putative genes. The availability of the draft genome sequence heralded pigeonpea improvement initiatives, setting a foundational step for future precision breeding efforts in the crop (Varshney *et al*., 2012). Building on this momentum, Professor Varshney continued his trailblazing contributions to genomics by leading sequencing initiatives for other important crops like chickpea (Varshney *et al*., 2013), and the A‐progenitor of groundnut (Chen *et al*., 2016). In 2017, the genome of pearl millet was decoded by his team, providing a resource to develop modern genetic solutions to support over 90 million farmers spanning sub‐Saharan Africa, India, and South Asia. The pearl millet genome (Tift 23D2B1‐P1‐P5) spanned approximately 1.79 Gb and was estimated to harbour 38 579 protein‐coding genes. The expansion/contraction analysis of the predicted genes highlighted substantial enrichment for wax biosynthesis genes, which may contribute to heat and drought tolerance in pearl millet (Varshney *et al*., 2017a). Beyond the initial sequencing efforts, his team also utilized the state‐of‐the‐art Hi‐C sequencing approach to add another layer of refinement to these genomes, thereby increasing the accuracy and applicability of these genomes (Garg *et al*., 2022). Professor Varshney stands at the forefront of implementing advanced technologies. In one of his collaborative endeavours, near‐gapless genome assemblies of two widely used soybean cultivars (Williams82 and Lee) were reported using long‐read sequencing technologies (Garg *et al*., 2023a). All the genomic resources generated under Professor Varshney's leadership are publicly available, serving as a rich resource for the global scientific community. These resources have been integrated into breeding programmes worldwide, leading to the development and deployment of crop varieties with improved yields, better disease resistance, and greater adaptability to diverse environmental conditions.

### Genome diversity catalogues

Following the successful decoding of reference genomes, Professor Varshney focused his efforts on resequencing projects, firmly establishing himself at the forefront of legume genomics. These efforts were aimed not only to unearth the rich genetic makeup of these important crops but also to understand how genetic variation could be harnessed to address pressing agronomic challenges. In the case of pigeonpea, whole genome resequencing (WGRS) of 292 diverse *Cajanus* accessions, including breeding lines, landraces, and wild species, led to the identification of 17.2 million variations (15.1 million SNPs, 0.9 million small insertions, and 1.2 million small deletions). By demonstrating the Central Indian state of Madhya Pradesh as the likely centre of origin of pigeonpea, the study helped resolve the long‐standing ambiguity surrounding the centre of origin of the crop. The GWAS analysis of these variations highlighted the genomic regions affected by domestication and pinpointed loci associated with phenotypic variation for agronomically important traits relevant to pigeonpea breeding (Varshney *et al*., 2017b). Turning their attention to chickpea, Professor Varshney and his group re‐sequenced 429 lines from 45 countries to generate a variation map of 4.9 million SNPs. This study provided: (a) discernible signs of selection during chickpea breeding, (b) Eastern Mediterranean as the primary centre of origin of chickpea, and (c) marker‐trait associations and candidate genes, including TIC, REF6, aspartic protease, cc‐NBS‐LRR, and RGA3, for drought and heat tolerance (Varshney *et al*., 2019a). Subsequently, Professor Varshney led a team of 57 researchers from 41 organizations across 10 countries to further investigate the genetics of chickpea. The outcome of this work was a chickpea pangenome (592.28 Mb), developed from the genome sequencing data from 3366 diverse chickpea lines (encompassing 3171 cultivated and 195 wild chickpea accessions) spanning 60 countries. The analysis provided details on chickpea's origin and migration routes to various parts of the world, the divergence time of different chickpea species, and the genetic loads/burdens responsible for lowering crop performance. The study also provided superior haplotypes for agronomic traits for undertaking haplotype‐based breeding and laid a foundation for genomic prediction and optimal contribution selection for developing superior varieties (Varshney *et al*., 2021a). This landmark study not only received recognition within the scientific community, but received broader attention from an interested public, featuring in prominent international media outlets, e.g., *The New York Times*, *The Economist*, and the BBC.

### High‐throughput and cost‐effective genotyping platforms

The deployment of genomic resources within breeding programmes has been mostly constrained by technological limitations and high costs associated with genotyping. To harness the extensive genomic resources for breeding purposes, there was a critical need for cost‐effective, high‐throughput genotyping platforms to develop dense genetic maps and conduct QTL analysis. SNP genotyping platforms serve multiple purposes, including estimating genetic diversity, fine mapping, association mapping, foreground selection, genomic selection, and evolutionary research. Professor Varshney and his team at ICRISAT addressed this need by developing low‐, medium‐, and high‐density SNP arrays in three legume crops: chickpea, pigeonpea, and groundnut. Notable examples include the development of Affymetrix arrays like the Axiom®*CicerSNP* Array for chickpea (Roorkiwal *et al*., 2018), Axiom®*CajanusSNP* Array for pigeonpea (Saxena *et al*., 2018), and the Axiom®*ArachisSNP* for groundnut (Pandey *et al*., 2017a), encompassing up to 58 000 SNPs. As a snapshot of the genome‐wide variations, these arrays have laid a robust foundation for high‐throughput genotyping, proving vital for foundational research and practical breeding applications. Additionally, to conduct cost‐effective genotyping for implementing Genomic Selection (GS) in legume breeding, 5000 SNP panels were developed for chickpea and groundnut through a targeted sequencing methodology. To incorporate target SNPs into breeding programmes, GoldenGate and VeraCode assays were also devised to genotype several hundred SNPs in crops such as chickpea and pigeonpea (Roorkiwal *et al*., 2013; Varshney, 2016).

Genotyping with high‐density arrays can incur significant costs, especially when genotyping large breeding populations for purposes like marker‐assisted selection (MAS), backcross breeding, and QC (quality control) of parental analysis. In applications where only a few markers are needed, cost‐effective and high‐throughput genotyping platforms are more suitable. To address this need, Professor Varshney, in collaboration with the International Maize and Wheat Improvement Center (CIMMYT) and International Rice Research Institute (IRRI), formulated a proposal that successfully secured $4 million in funding from the Bill & Melinda Gates Foundation. The High Throughput Genotyping (HTPG) project offered low‐cost genotyping services at ~$1.5 per sample for genotyping with 10 SNPs, including DNA extraction (https://cegsb.icrisat.org/high‐throughput‐genotyping‐project‐htpg/). This service now encompasses over 1000 SNPs for 100+ traits and 500+ QC SNPs. In partnership with Intertek, the project extended its services to eight CGIAR centres, 30+ NARS, and four private companies in more than 28 countries, covering over 18 crops (Bohar *et al*., 2020). Notably, the project significantly reduced costs for CGIAR and NARS by generating genotyping data at only a fraction (approximately one‐third to one‐fourth) of the original price. Furthermore, Professor Varshney also spearheaded efforts to develop cost‐effective, 10 SNP panels in chickpea, pigeonpea, and groundnut to facilitate early‐generation selection in breeding programs (Varshney *et al*., 2018). For instance, single‐plex, cost‐effective platforms like Kompetitive Allele Specific PCR (KASP) assays are key for SNP genotyping in MAS applications. KASP assays have been developed for several thousand SNPs in chickpea, pigeonpea, and groundnut (Hiremath *et al*., 2012; Khera *et al*., 2013; Saxena *et al*., 2012). Some of these markers are currently being utilized as diagnostic indicators for critical traits, such as drought tolerance in chickpea (Barmukh *et al*., 2022a,b), high oleic acid and foliar fungal disease resistance in groundnut (Pandey *et al*., 2023) within breeding programmes.

### Gene expression atlases for legume crops

Under the guidance of Professor Varshney, advanced technologies like transcriptome sequencing and bioinformatics tools have been adeptly utilized to study the intricate patterns of gene expression across various tissues, developmental stages, and biotic/abiotic conditions (Garg *et al*., 2023b). Analysis of these patterns, by Professor Varshney's team has unravelled the intricate genetic interactions that underpin vital physiological processes in legumes such as chickpea, pigeonpea, and groundnut. Focusing on chickpea, his team developed the *Cicer arietinum* Gene Expression Atlas (CaGEA) using the drought‐resistant cultivar ICC 4958. A total of 816 million raw reads were obtained from 27 tissues spanning five major developmental stages. The transcriptome data analysis pinpointed genes that play a role in flowering, nodulation, and the development of seeds and roots (Kudapa *et al*., 2018). Similarly, in pigeonpea, he led the development of the *Cajanus cajan* gene expression atlas. This atlas facilitated the identification of two regulatory genes, a pollen‐specific SF3 and a sucrose–proton symporter, with implications for improving agronomic traits, e.g., seed production and yield (Pazhamala *et al*., 2017). To understand the development process of groundnut, his team developed the *Arachis hypogaea* gene expression atlas (AhGEA) for the world's widest cultivated subsp. *fastigiata*. AhGEA unveiled mechanisms behind complex regulatory networks, including gravitropism and photomorphogenesis, seed development, and oil biosynthesis in groundnut. Additionally, the analysis identified candidate genes associated with allergens, which following functional validation, might be used to develop allergy‐free, consumer‐friendly groundnut varieties (Sinha *et al*., 2020).

### Multi‐omics for understanding stress responses

Understanding the intricate layers of biological systems demands a multi‐modal approach. The integration of multi‐omics approaches allows researchers to achieve a holistic perspective, unlocking the synergies between genes, transcripts, proteins, and metabolites. In this direction, Professor Varshney and his team employed multi‐omics approaches to delineate the stress response in different crops. As an example, his team employed transcriptome, small RNA, and degradome sequencing to understand the resistance mechanism of chickpea against Ascochyta blight (AB), a significant factor limiting global chickpea production. Transcriptome sequencing identified 6767 DEGs, primarily linked to disease resistance, pathogenesis‐related proteins, and cell wall biosynthesis. Small RNA sequencing pinpointed 651 miRNAs, with 297 exhibiting differential expression across genotypes and conditions. Integrating small RNA and transcriptome data revealed 12 contrasting miRNA–mRNA interaction pairs in resistant and susceptible genotypes, shedding light on the genes potentially involved in AB infection (Garg *et al*., 2019). Similarly, his team used transcriptomics, proteomics, and metabolomics to unravel complex mechanisms regulating drought response in chickpea. The integrated root‐omics data identified key proteins (encoding isoflavone 4′‐O‐methyltransferase, UDP‐d‐glucose/UDP‐d‐galactose 4‐epimerase, and delta‐1‐pyrroline‐5‐carboxylate synthetase) and metabolites (fructose, galactose, glucose, myoinositol, galactinol, and raffinose) linked to various pathways crucial for drought response (Kudapa *et al*., 2023).

Professor Varshney's group has also led and collaborated with partners from USA to develop mitigation approaches to reduce aflatoxin contamination in groundnut, using transcriptomics to explore both host‐pathogen interactions and the responses of *Aspergillus flavus* to drought‐related stresses. For example, Professor Varshney's group examined the transcription responses of seven groundnut lines with reduced aflatoxin contamination to *A. flavus* infection along with one highly susceptible line. This work identified several differentially expressed genes potentially associated with reduced aflatoxin including transcription factors, pathogenesis‐related proteins, glutathione‐S‐transferases, resveratrol synthase, and chitinase (Soni *et al*., 2021). Additional transcriptome studies on the *A. flavus* pathogen were also carried out with Professor Varshney's group in collaboration with the USDA‐ARS and the University of Georgia. These efforts resulted in the transcriptome sequencing of 62 RNA‐seq libraries from six different *A. flavus* isolates with varying levels of aflatoxin production capabilities describing their responses to drought‐related oxidative stress (Fountain *et al*., 2016a,b, 2020a). This identified a major role of fungal secondary metabolism, including aflatoxin production, in oxidative stress responses of this pathogen. This and other follow‐up studies also informed the selection of isolates for use in the development of new chromosome‐arm reference genome assemblies for comparative analyses in *A. flavus* (Fountain *et al*., 2020b).

### Genetics maps and marker‐trait associations

Over the past few decades, there has been a notable surge in the development of extensive genomic resources in legumes such as chickpea, pigeonpea, and groundnut. This progress can be attributed to the dedicated efforts of Professor Varshney and his team at ICRISAT. The group was crucial in generating several thousand SSRs and diversity array technology (DArT) markers for each of the three legume crops (Varshney, 2016). Additionally, in recent years, a substantial number of SNP markers, which were previously lacking in these legume crops until 2005, have been successfully developed, numbering in millions. This large‐scale marker information, combined with the development of cost‐effective marker assays (as mentioned above), was subsequently employed for trait mapping and gene discovery in the targeted crops.

To maximize the utility of genomic resources in breeding efforts, Professor Varshney and his team conducted extensive trait mapping and developed over 50 genetic maps in various legume crops, particularly chickpea, pigeonpea, and groundnut. Using linkage mapping and association mapping approaches, they mapped more than 30–50 traits in each of the three legume crops. For example, to unravel the intricate nature of drought tolerance in chickpea and pinpoint markers associated with drought tolerance‐associated traits, Professor Varshney led efforts in identifying a “*QTL‐hotspot*” region on linkage group 4, harbouring 12 main‐effect QTLs for drought tolerance‐related traits, accounting for up to 58.20% of phenotypic variation (Kale *et al*., 2015; Varshney *et al*., 2014). Further, Professor Varshney and colleagues successfully identified genomic regions/candidate genes for resistance to sterility mosaic disease (Gnanesh *et al*., 2011), Fusarium wilt (Saxena *et al*., 2017), and various abiotic stresses that pose substantial yield constraints in pigeonpea. In the case of groundnut, his team successfully mapped QTLs associated with resistance to rust and late leaf spot (Pandey *et al*., 2017b), oil content (Shasidhar *et al*., 2017), and yield‐related traits (Pandey *et al*., 2020; Vishwakarma *et al*., 2017).

## Novel concepts and genomic innovations for agricultural metamorphosis

Professor Varshney has been at the forefront of introducing and advancing several pivotal concepts and methodologies that have fundamentally transformed the landscape of crop improvement through genomics. His contributions have significantly enriched the field of agricultural genetics and breeding, ushering in new paradigms and approaches.

### Genomics‐assisted breeding

In 2005, Professor Varshney introduced the concept of “Genomics‐assisted breeding” (GAB) in the 10th Anniversary Issue of Trends in Plant Science, titled “Feeding the World: Plant Biotechnology Milestones” (Varshney *et al*., 2005b). GAB revolutionized crop improvement by harnessing cutting‐edge genomic tools, such as functional molecular markers, advanced bioinformatics, and enhanced knowledge of statistics and inheritance patterns. This approach significantly increased the efficiency and precision of crop improvement across the world. It was envisioned that GAB would be a transformative force in developing and disseminating improved crop varieties, including those with high yields and resilience against pests, diseases, and environmental stresses. The present‐day success stories based on the improved crop varieties resulting from GAB are a testament to this vision. GAB has accelerated the pace of breeding progress across a diverse range of crop species, developing over 130 publicly bred cultivars worldwide (Varshney *et al*., 2021b). Notably, GAB has been pivotal in producing improved cultivars with heightened resistance to key diseases like bacterial blight and blast in rice, rust in wheat, and fusarium wilt in chickpea. Significant progress has been made in improving abiotic stress adaptation (such as tolerance against submergence, salinity, and drought) and enhanced nutritional quality (e.g., wheat varieties with higher grain protein content, groundnut varieties with elevated oleic acid content, intermediate amylose content in rice varieties, as well as quality protein maize cultivars) in different crops using GAB.

### The next chapter: Genomics‐assisted breeding 2.0

Recent innovations in genome sequencing, precise phenotyping, genetic diversity analysis, and genome editing technologies offer significant potential for identifying and aggregating superior alleles for target traits in crop improvement. Acknowledging this, in the 25th Anniversary Issue of Trends in Plant Science, themed “Feeding the World: The Future of Plant Breeding”, Professor Varshney introduced a comprehensive approach, referred to as “genomics‐assisted breeding 2.0” (GAB 2.0) or “genomic breeding”, for shaping future crops (Varshney *et al*., 2021b). It involves fine‐tuning crop genomes by accumulating advantageous alleles and removing detrimental ones to design future crops. In the years ahead, GAB 2.0 will help devise efficient strategies to breed climate‐resilient crop varieties with high nutritional value while upholding sustainability and environmental conservation.

### Super‐pangenome

A pangenome represents the complete set of genes found within a particular species, capturing the full spectrum of its genetic diversity. This vast genetic reservoir is pivotal for crop improvement as it allows researchers to identify beneficial alleles or allele combinations that can be harnessed to enhance crop traits. Traditionally, pangenomes were constructed mainly from the cultivated gene pools of a specific species, occasionally incorporating two or three closely related species. However, recognizing that a crop's gene pool includes a multitude of species, especially wild relatives with diverse genetic makeup, Professor Varshney and his team proposed extending the pangenome approach beyond cultivated pool by including accessions from all available species within a genus. The resulting “Super‐Pangenome” traverses the full landscape of crop diversity for rapid and transformative crop improvement (Khan *et al*., 2020). Within a short span of time, this new approach has spurred a growing number of published literatures on building super‐pangenomes in various crops including tomato, potato, and rice (Raza *et al*., 2023).

### 5Gs for crop genetic improvement

Professor Varshney and his collaborators coined the 5G paradigm that calls for the need to make the best use of genome assemblies, germplasm characterization, gene function elucidation, genomic breeding, and gene editing for crop improvement (Varshney *et al*., 2020). Highlighting the imperative of robust genome assemblies and in‐depth germplasm characterization, he advocates for sequencing‐based identification of accurate breeding targets to enable optimized genomic breeding and gene‐editing methodologies. Although elements of the 5Gs are sporadically employed in global crop improvement initiatives, comprehensive 5G integration remains elusive, particularly in developing world. Professor Varshney and his collaborators underscored the potential of emerging technologies in sequencing, phenotyping, and data science to catalyse the global adoption of the 5G strategy and suggested that a fully realized 5G approach can significantly bolster breeding precision, yielding climate‐resilient, nutritious varieties with accelerated genetic gains.

### Fast‐forward breeding

To meet the demands of a rapidly growing global population, agricultural systems around the world must increase their outputs in a sustainable manner. Considering this challenge, Professor Varshney and colleagues introduced the “Fast‐forward breeding” framework for accelerated crop improvement (Varshney *et al*., 2021c). This framework offers a comprehensive strategy for incorporating cutting‐edge technologies in crop genome sequencing, high‐throughput phenotyping, and systems biology, together with efficient trait mapping and genomic prediction, including machine learning and artificial intelligence, to expedite the availability of advantageous traits for breeding and research purposes. Approaches like haplotype‐based breeding, genomic prediction, and genome editing, outlined in this framework, are anticipated to hasten the targeted integration of superior genetic traits into future cultivars. Additionally, emerging breeding techniques, such as optimal contribution selection, can enrich the genetic diversity of breeding programs while accelerating genetic advancements. Combining speed breeding with state‐of‐the‐art genomic breeding technologies has the potential to overcome the longstanding bottleneck of protracted crop breeding cycles. The methods outlined in this framework and their integration are poised to accelerate the breeding process for enhanced crop improvement, ultimately contributing to a more food‐secure world.

## Transformation of smallholder agriculture

With an objective to implement GAB in breeding programmes of chickpea, pigeonpea, and groundnut, Professor Varshney trained >500 breeders from 36 countries from Asia, Africa and South America and provided them access to high throughput and cost effective genotyping platforms. This collaboration enabled several Asian and African breeding programmes to successfully incorporate GAB, leading to development of numerous improved lines. Following stringent agronomic evaluations, many of these improved lines were recommended and delivered to farmers and several are in the advanced stages of the varietal release processes in countries like India, Ethiopia, Kenya, Tanzania, Ghana, Mali, and others. For example, in the case of chickpea, introducing the “*QTL‐hotspot*” region through the GAB approach led to the development and release of several drought‐tolerant varieties for cultivation in India and Ethiopia over the past few years. This list of improved varieties includes Pusa Chickpea 10216, Pusa Chickpea 4005, IPC L4‐14, Pusa Chickpea Shyam, and Geletu, among others (for details see Roorkiwal *et al*., 2020). Particularly noteworthy is “Pusa Chickpea 10216”, the first GAB‐led chickpea variety in India with enhanced drought tolerance and an 11% yield advantage over the recurrent parent (Bharadwaj *et al*., 2021). Similarly, several Fusarium wilt resistant chickpea varieties, namely, Super Annigeri 1, Samriddhi, Pusa Manav, were delivered to chickpea farmers in India. In pigeonpea, Professor Varshney and team contributed to the development of low‐cost and rapid molecular marker assays to test the genetic purity of hybrids and parental lines along with genomic prediction tools to obtain high heterotic combinations, thus paving the way for a full‐scale commercial hybrid breeding technology in pigeonpea (Bohra *et al*., 2020; Saxena *et al*., 2021). In the case of groundnut, high oleic groundnut varieties, specifically “Girnar 4” and “Girnar 5”, featuring nearly 80% oleic acid content compared to the typical varieties with 40–50% oleic acid content, were developed through a collaboration between ICAR‐Directorate of Groundnut Research in Junagadh and Professor Varshney's team at ICRISAT (Pandey *et al*., 2020). Furthermore, Professor Varshney also played a key role in the marker‐assisted improvement of popular groundnut varieties for resistance to rust and late leaf spot. Several of these lines are in advanced stage of public release in India and many countries in Africa. These high‐yielding lines, with enhanced biotic/abiotic stress tolerance, underscore the potential of integrating genomics with breeding endeavours to develop superior, climate‐resilient varieties.

With an objective of creating a positive difference in the livelihoods of smallholder farmers in Sub‐Saharan Africa and Asia, Professor Varshney led “Tropical Legumes III” (TL III, https://tropicallegumeshub.com/) project as a Principal Investigator, with a total budget of USD 25 million from the Bill & Melinda Gates Foundation (Varshney *et al*., 2019b). Through collaboration with scientists from three CGIAR Centres (ICRISAT, IITA, CIAT) and 15 national programmes in target countries, TL III strategically targeted four crops – cowpea, common bean, groundnut, and chickpea – across seven regions, including Burkina Faso, Ghana, Mali, Nigeria, Ethiopia, Tanzania, Uganda, and the Indian state of Uttar Pradesh. Professor Varshney played a key role in providing strategic guidance and oversight for the development and accessibility of high‐yielding varieties for farmers. TL III and its predecessor (TL II Phase I and Phase II projects), led directly by Professor Varshney, played a pivotal role in releasing 266 varieties, producing 497 901 tons of certified seeds that were planted on about 5.0 million ha in the 15 countries and beyond, producing about 6.1 million tons of grain worth USD 3.2 billion (Ojiewo *et al*., 2019). The project outputs have benefitted >23 million lives, especially women and children. As per an independent economic analysis by Evans School Policy Analysis and Research of the University of Washington, the average benefit‐to‐cost ratio for this project is 16 indicating massive social returns the project delivered. In recognition of this remarkable work and its positive effects on the livelihoods of smallholder farmers in 13 African countries, Professor Varshney's previous organization, ICRISAT was honoured with the Africa Food Prize in 2021.

## Growing the crop science community by mentoring and empowering researchers

Professor Varshney's commitment to advancing agricultural research extends beyond his scientific endeavours. He has played a pivotal role in capacity building and mentoring the next generation of researchers and agricultural leaders. Delivering 15 training courses, attended by more than 500 breeders from 36 different countries, Professor Varshney has shared his wealth of knowledge and expertise with aspiring scientists, particularly from developing countries. Additionally, he has organized/organizing several conferences and workshops, including 3rd International Wheat Congress (2024), Plant & Animal Genome Conference – Australia (2023), 5 editions of Next Generation Genomics & Integrated Breeding for Crop Improvement, 15th ADNAT Convention on Genomics and Biodiversity, InterDrought‐V Conference (2017), International Congress on Legume Genetics and Genomics (2012) and Genomics‐Assisted Breeding workshop annually in PAG USA and many more. These workshops/conferences have served as platforms for fostering collaboration, exchanging ideas, and disseminating the latest advancements in genomics‐assisted breeding and crop improvement. His unwavering dedication to nurturing talent and providing access to cutting‐edge techniques has empowered countless individuals to contribute to the field of genomics and agriculture.

Notably, Professor Varshney has also supervised nearly 50 PhD students and 70 post‐docs many of whom have gone on to establish successful careers in their respective fields. His mentorship has not only shaped careers but has also been instrumental in strengthening the global research community's capacity to address pressing agricultural challenges. His legacy of mentorship and capacity building continues to inspire and guide emerging scientists in their pursuit of scientific excellence.

By serving several organizations in the science leadership/management role for over 15 years, Professor Varshney has supervised large international teams representing a range of diversity in ethnicity, nationality, and gender. In these roles, Professor Varshney offered extensive proficiency in R&D management, product development, fostering innovative ideas, and championing an inclusive culture. He also actively encouraged an inter‐disciplinary and collaborative research environment, empowering team members to achieve high‐performance and unlock their full potential as both team contributors and individual achievers.

Due to his outstanding science contributions and his impact‐oriented research, Professor Varshney has remained a sought‐after research leader to be invited to various committees and agencies across the globe. He has served/serving several organizations in various roles, including (a) Board of Governors: International Crop Science Society, (b) Board of Directors: DivSeek International, (c) Board Members: DivSeek, International School of Hyderabad, (d) Academy/Society Membership: Royal Society (Sectional Committee), Crop Science Society of America (C7 Division Chair), National Academy of Agricultural Sciences (Foreign Secretary, Executive Council), Indian National Science Academy (Sectional Committee), (e) Research Advisory Council Member: DBT – National Agri‐Food Biotechnology Institute (NABI), India; DBT – Center of Innovative and Applied Bioprocessing (CIAB), India; ICAR – National Institute of Plant Biotechnology, India; Science and Engineering Research Board of Department of Science and Technology, India. Professor Varshney's exemplary role across these scientific communities encapsulates his commitment to fostering innovation and sustainability in crop science, benefiting countless stakeholders in the agri‐food sector worldwide.

Professor Varshney's extensive contributions to academia extend beyond his impactful research. He has been instrumental in shaping scientific discourse through his longstanding editorial roles in numerous prestigious journals. With over a decade at the *Plant Biotechnology Journal*, including more than 4 years as a Senior Editor, and editorial positions in key journals such as *Trends in Plant Sciences*, *Plant & Cell Physiology*, *Theoretical and Applied Genetics*, *Plant Breeding*, *Frontiers in Plant Science*, *Molecular Genetics and Genomics*, *The Plant Genome*, and *Plant Genetic Resources*, he has helped to steer the direction of plant science research.

## Inspiring recognitions and honours throughout an illustrious journey

**Figure 4:**
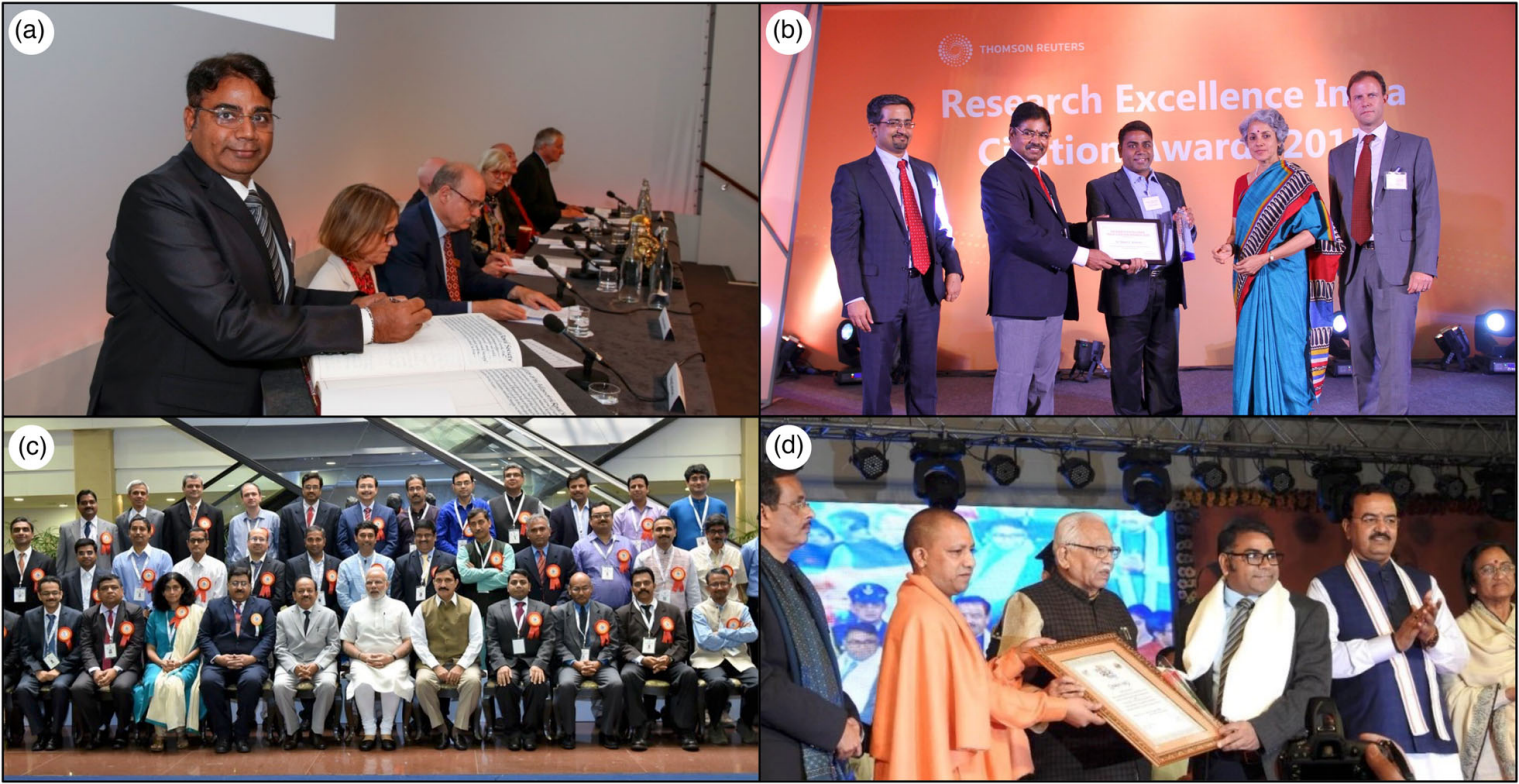
Recognition of Professor Varshney at select prestigious award ceremonies. (a) Signing the 366‐year‐old Royal Society Charter during his induction as a Fellow of the Royal Society (2023); (b) receiving the Research Excellence India Citation Award from Thomson Reuters (2015); (c) in the group photo with the Prime Minister of India, Mr Narendra Modi, along with some other awardees of the Shanti Swarup Bhatnagar Prize (2015); and (d) being honoured by the Chief Minister, the Government of Uttar Pradesh (home state), India, Mr Yogi Adityanath (2018).

**Figure 5:**
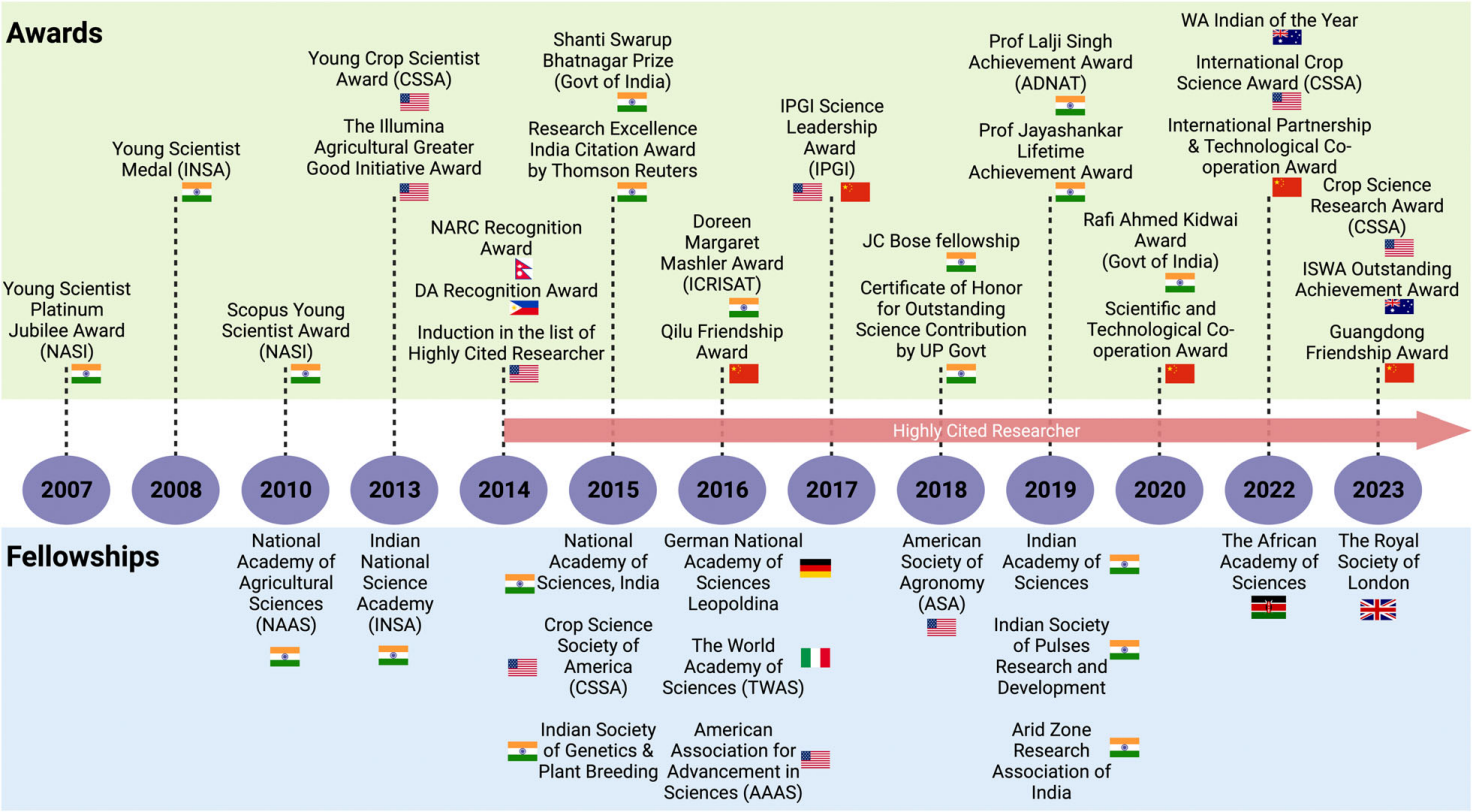
A timeline highlighting select accolades and fellowships presented to Professor Varshney for his significant research contributions.

Professor Varshney's pioneering research contributions have been recognized and celebrated nationally and internationally throughout his illustrious career. His accolades are a testament to his dedication, innovation, and work's profound impact (Figures 4 and 5). His esteemed standing is evident from his election as a fellow to more than 10 esteemed science and agriculture academies/societies spanning India, Germany, the USA, the UK, Italy, and Africa. His work has earned him over 40 prestigious awards from different countries, including the Shanti Swarup Bhatnagar Prize and the Rafi Ahmed Kidwai, India's pinnacle of science and agricultural science awards. Professor Varshney has been invited to share his expertise at several esteemed international conferences, delivering presentations at high‐profile meetings, including Plenary talk at the 30th Plant and Animal Genome (PAG) conference, USA (the world's largest genomics conference) in 2023; G8 International Conference on Open Data for Agriculture on Open Data in Genomics and Modern Breeding for Crop Improvement, organized by US and UK governments in the World Bank, 2013; brainstorming session on Digital Revolution for Agriculture at Bill & Melinda Gates Foundation in 2012, chaired by Mr Bill Gates, co‐chair of Bill & Melinda Gates Foundation; FAO's international conference on Agricultural Biotechnology in Developing Countries in Mexico in 2010 and FAO's Regional Conference on Agricultural Biotechnologies in Sustainable Food Systems and Nutrition in Malaysia in 2017.

## Disseminating scientific knowledge and communication to society

With an objective of advancing plant biology and agricultural science, Professor Varshney, since his master's degree, has been proactive in publishing scientific articles. He is a prolific scientific author and has published a large number of papers (>600) in various high‐impact journals including *Nature*, *Nature Genetics*, *Nature Biotechnology*, *Plant Biotechnology Journal*, *Trends in Plant Science*, *Proceedings of the National Academy of Sciences, USA*, *Molecular Plant*, *New Phytologist*, *Plant, Cell & Environment*, and *Journal of Experimental Botany*. As of December 16, 2023, Professor Varshney boasts an impressive h‐index of 124, with his work cited over 66 000 times. Due to the substantial impact of his research, Clarivate Analytics has consistently recognized him as a “Highly Cited Researcher” since 2014.

Professor Varshney is not only a luminary in the scientific world of genomics and agriculture but also an ardent advocate for bridging the divide between scientific research and the public. Acknowledging the significance of making science both accessible and relatable, he has persistently strived to convey intricate scientific concepts in ways that resonate with a broader audience. A prime example of his outreach is his presentation on the TEDx stage, where his talk illuminated the complexities of genomics and its agricultural implications, making the topic both captivating and comprehensible to those outside the scientific community. Beyond public speaking, Professor Varshney's research has been featured in numerous international print and electronic media outlets, including *The New York Times*, *The Economist*, *Forbes*, BBC, ABC, Cosmos, Down to Earth, Mongabay, Doordarshan, and many others. His insights are highly sought after, and he utilizes these platforms to highlight the latest genomic advancements, their potential applications, and the forthcoming challenges. Embracing the digital era, Professor Varshney effectively utilizes online platforms to engage a wider audience. He maintains an active YouTube channel (https://www.youtube.com/@rajeevvarshney6803) to highlight videos that explore various facets of genomics and his research initiatives. Additionally, his commitment to effective communication is evident in his written contributions to his blog (https://rajeevkvarshney.wordpress.com/), where he regularly articulates insights into the scientific realm. Through his videos and articles, he imparts recent research developments and shares personal reflections on the odyssey of discovery. These platforms serve not merely as knowledge repositories but as catalysts that ignite curiosity, inspiring young enthusiasts to delve into the world of scientific discovery.

## Personality beyond the sequences

While Professor Varshney's scientific accomplishments place him among the luminaries in legume genomics and agriculture, his persona is rich and multifaceted beyond academic achievements. Beyond the image of the distinguished researcher is a man with diverse interests, deeply engaged in his work and the wider world. A fervent reader, Professor Varshney's curiosity is not limited to his laboratory. His office shelves are adorned not only with scientific journals but with a myriad of books spanning across genres. This reading habit undoubtedly nourishes his vast appetite for knowledge and broadens his understanding of diverse topics. However, his inquisitiveness is not just confined to books. A movie buff at heart, Professor Varshney enjoys delving into the world of cinema, appreciating the art of storytelling from various cultures and perspectives. This love for movies offers him a refreshing escape, a way to momentarily disconnect from the scientific rigours and immerse himself in narratives far removed from the world of genomics. Despite the demands of his profession, he never misses an opportunity to revel in moments of fun and leisure. His fun‐loving nature ensures that the atmosphere around him, whether at conferences or team meetings, is light‐hearted, promoting creativity and camaraderie among his colleagues and students. In considering these dimensions, we perceive Professor Varshney holistically: as a trailblazing scientist, an avid reader, a cinema aficionado, and a source of joy in the often‐demanding field of research. This equilibrium between professional dedication and diverse passions positions Professor Varshney as an inspiration inside and outside the laboratory.

## Conflict of interest

The authors declare no competing interests.

## Data Availability

Data sharing not applicable to this article as no datasets were generated or analysed during the current study.
